# Surgical Management of Breast Cancer in Africa: A Continent-Wide Review of Intervention Practices, Barriers to Care, and Adjuvant Therapy

**DOI:** 10.1200/JGO.2016.003095

**Published:** 2016-07-06

**Authors:** Stephanie A. Sutter, Aaron Slinker, Deo Darius Balumuka, Katrina B. Mitchell

**Affiliations:** **Stephanie A. Sutter**, Weill Cornell Medical College; **Aaron Slinker**, New York University, New York, NY; **Deo Darius Balumuka**, Mbarara University of Science and Technology, Mbarara, Uganda; and **Katrina B. Mitchell**, The University of Texas MD Anderson Cancer Center, Houston, TX.

## Abstract

Breast cancer has emerged as a leading cancer among women in Africa, necessitating improved understanding of its management across the continent. Although previous studies have described regional trends in therapy, this review aims to summarize continent-wide management and focus specifically on surgical interventions. Current literature shows that the rates of surgery, chemotherapy, and radiation therapy vary across different countries and institutions, indicating the need for greater use of standardized cancer treatment guidelines. Surgery, primarily modified radical mastectomy, is the most common form of therapy described. When chemotherapy is offered, the limited availability and cost of treatment lead to high rates of interruption and premature termination of cycles. Few patients have access to radiation or hormonal therapy because these treatments are not available in many countries. Significant delays in seeking treatment are common and contribute to patients presenting with advanced disease. Although limited infrastructure favors surgical management, interventions to improve early detection behavior, provide timely referrals to medical care, and initiate early treatment with access to clinically justified neo-adjuvant and adjuvant therapy are key to improving prognosis.

## INTRODUCTION

Breast cancer is a paramount concern to women’s health. It is the most common invasive cancer in the world; more than 1.4 million women receive the diagnosis of breast cancer every year.^1^ Management of this disease among Western health care systems has led to remarkable improvements in outcome in the past 40 years. In the United States, the spike in breast cancer incidence in the 1980s corresponded with a decrease in mortality because the widespread use of mammography as a screening tool detected more cases of cancer at an earlier stage of disease.^2^ As a result, the overall 5-year survival in the United States is now almost 92% and continues to rise.^2^

These improvements in breast cancer management do not reflect the current situation throughout the African continent, where women suffer a particular burden of disease and lack resources for detection and treatment. Estimates of cancer statistics released in 2012 for 26 African countries indicated that breast cancer has become a leading cancer among women.^3^ Of special concern, patients present late with advanced disease, treatment is often unavailable, delayed, or prohibitively expensive, and survival rates are low.^3^ Early research suggests that breast cancer among African women is more aggressive, with a greater proportion of patients presenting at an earlier age and with high-grade and receptor-negative tumors.^3,4^ A literature search was conducted using search terms “breast cancer” and “Africa.” Whereas other studies have described regional variation of the occurrence of breast cancer in Africa, the focus of this review is to summarize the current management of breast cancer throughout the entire continent of Africa, specifically attempting to investigate surgical procedure rates and barriers to surgical care.

## EARLY DETECTION AND SCREENING

Although the current literature does not provide consistent detailed information regarding surgical management of breast cancer in various African countries, common trends are apparent that may make surgery a secondary concern to ministries of health. Screening is lacking in all the countries with published literature in this area. As noted in other articles, most recently in an article by Kantelhardt et al,^3^ screening via mammography and the appropriate resources for proper follow-up of any results are not widely available in Africa.^3,5,6^ Thus, patients may present at a late stage, when palliative chemotherapy is of more use than surgery. Surgery may be used for palliative management only when the resources needed to provide sufficient chemotherapy are lacking or unavailable.^3^

From studies in Ghana, Nigeria, South Africa, Cameroon, Eritrea, Rwanda, Tanzania, and Uganda, only three countries report the main form of breast cancer detection, which is self-examination: Ghana (62.2%), Nigeria (92%, Ile-Ife; 73.3%, Enugu), and Cameroon (92.9%). ^7-10^ Only the Eritrea study notes the use of radiology in screening, and this is for only 29% of patients (Table 1). When an abnormality was detected via self-examination, a significant delay between detection and presentation for health care was reported. Of the 10 studies that note delay time, more than half of all patients waited 3 months or more to seek medical advice, with some averages approaching a year or more (Table 1). As a result, totals from 13 different studies show that more than 75% of patients presented in stages III to IV (Table 1). On the basis of this information alone, it is apparent that initiating programs that emphasize self-examination techniques and early access to care would be a major first step in making a proper place for the use of surgery and improving outcomes overall.

**Table 1:**
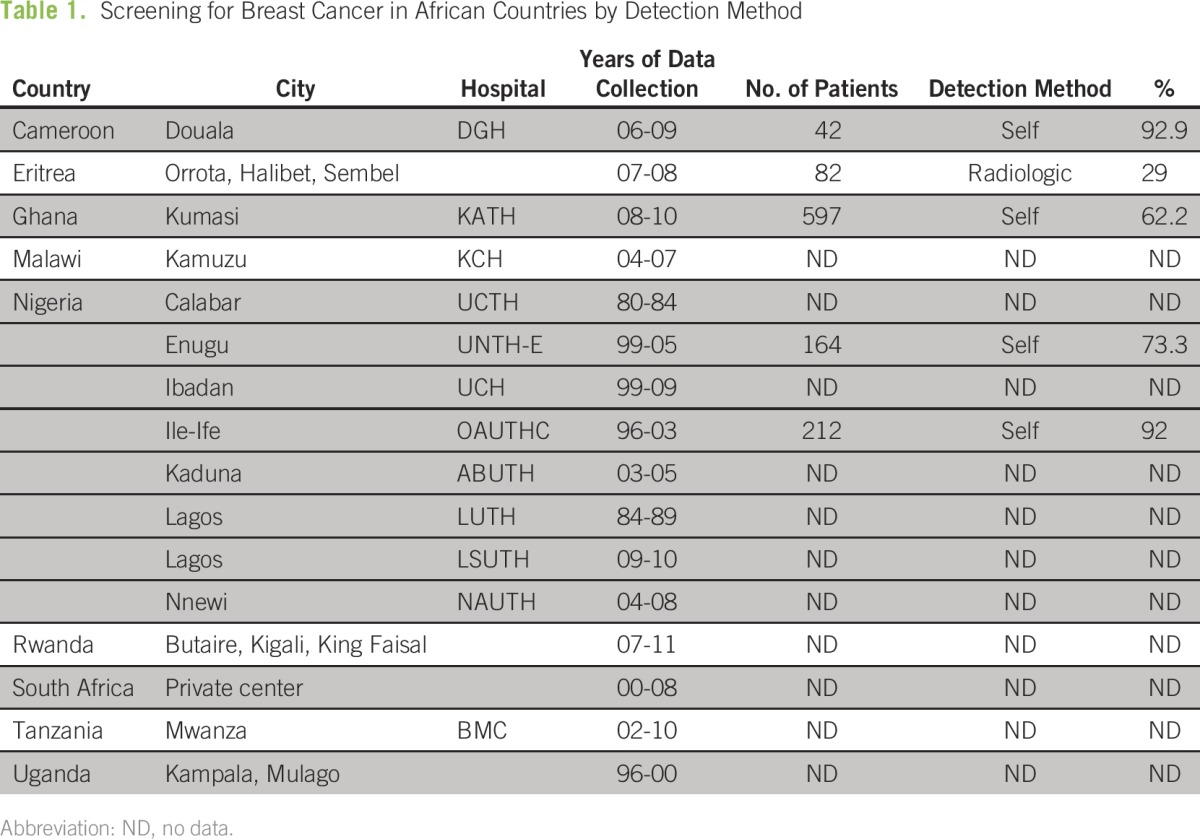
Screening for Breast Cancer in African Countries by Detection Method

The main causes of delay are attributed to the patients not understanding the severity of their disease and seeking treatment with alternative healers before presenting for medical attention. Patients commonly said that they were unaware of the implication of their disease or believed that it would disappear without treatment.^9,11^ Studies from Ghana, Eritrea, and Nigeria found that between 12.2% and 38.4% of patients initially sought treatment with an alternative practitioner, including traditional healers, prayer homes, and herbal specialists.^7,9,11-13^ Seeing an alternative practitioner was significantly associated with a delay of more than 3 months.^9^ Considering the relative scarcity of health care providers, this preference for traditional medicine is not surprising, because traditional healers are more affordable, are easily accessible, provide less invasive treatment, and offer a more intimate relationship.^7^ Therefore, it has been suggested that natural healers could be integrated into the medical framework and could provide patient referrals to hospitals, which would allow traditional and medical approaches to be implemented in parallel.^6^ Hospital-side delays also play a factor, with one study showing delayed or no referrals for treatment, erroneous advice from initial health care providers, delays in biopsy analysis, and infrastructural insufficiencies affecting 46.2% of patients.^9^ Finally, lack of funds is also cited as a barrier to seeking initial treatment, with approximately 3% of patients reporting this as the cause of their delay; however, prohibitive cost seems to be a greater factor in the interruption and discontinuation of treatment later in the course of breast cancer care.^11,14,15^

## SURGERY

Surgical intervention is the primary focus of treatment in Africa.^6^ Because tumor staging is rare in this region, most of the studies included in this review do not report treatment as guided by disease stage. Instead, therapy choice seems to be dictated by the local availability of resources; surgery represents the most popular option when access to chemotherapy and radiotherapy is limited. A need for more detailed record keeping throughout the continent is essential to understanding treatment decision making and the associated outcomes.

The rates of surgical treatment vary greatly across the different countries in Africa, ranging from 35.2% in Nigeria to 100% in Cameroon; the majority of countries report surgical rates between 48% and 75%^6,7,10,14-16^ (Table 2). This divergence in surgical treatment popularity seems to be dependent on both patient preference and country-specific resources. In Nigeria, for example, the comparatively low breast cancer surgery rate was attributable to inoperable advanced tumors, inability to pay for treatment, and patient unwillingness to have a mastectomy.^16^ In Eritrea, 80 of the 82 women who were observed received surgical intervention as their only treatment because there are no chemotherapy or radiotherapy options available nationally.^12^ Nguefack et al^10^ in Cameroon observed 42 women for breast cancer over a 3-year period, and nearly all were treated with some form of surgery (92.9%), despite the majority of patients presenting with stage III disease; 79% of patients received neoadjuvant chemotherapy to downstage tumors and therefore allow surgical excision and closure to occur.^10^ This suggests a possible regional difference in approach to multidisciplinary breast cancer care, as well as a chasm in cultural attitudes toward surgical options, because very few women in Eritrea and Cameroon are refusing mastectomies in comparison with their counterparts in Nigeria.

**Table 2:**
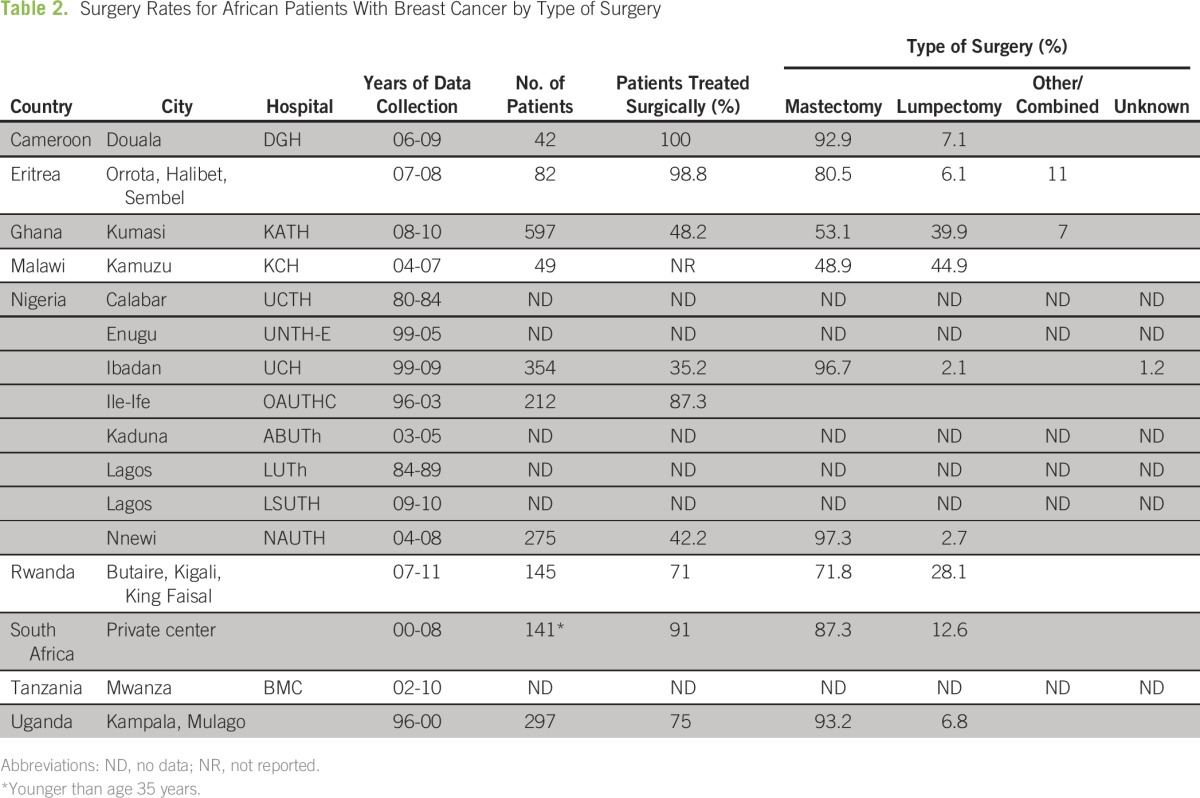
Surgery Rates for African Patients With Breast Cancer by Type of Surgery

When surgical options are pursued, the most common procedure is a mastectomy, with all institutions surveyed reporting a rate of well above 50%, with many above 90%.^7,10,16,17^ Modified radical mastectomy is most frequently performed, which consists of removing the whole breast tissue along with the axillary tail and complete clearance of axillary lymph nodes and connective tissue.^15,16^ Breast-conserving surgeries and lumpectomies remain rare in this region, because the majority of patients present with advanced stages (III and IV) of invasive cancer, which limits the options for conservative surgery in the absence of neoadjuvant chemotherapy.^6,8,15,17^ Furthermore, the ability to assess surgical margins for tumor-free tissue is often not feasible, and postoperative follow-up is low.^12,16^

Despite a shortage of other treatment options, many African women report fear of mastectomy, which may contribute to the delay in seeking surgical evaluation.^7,9,13,18^ Ajekigbe et al^18^ report that the most prevalent cause of delay among Nigerian women is fear of mastectomy; 44.7% of women surveyed acknowledged that they were aware that their ailment could be cancer, but they delayed treatment because of fear of mastectomy. This fear was a consistent cause of delay across age and educational background groups. Similar studies found that patients refuse mastectomy because they do not want their bodies to be disfigured and fear the effect of mastectomy on their relationship with their partners.^13^ A reason cited for treatment refusal is that family members believe that hospital treatment could result in an escalation of the disease.^13^ This fear of surgery and delay in treatment creates a cycle that may result in overall poor outcomes for the African region: when patients have a fear of mastectomy, they delay their treatment and seek alternative therapies, only presenting to the hospital when the disease has escalated to an advanced stage. At advanced stages, aggressive treatment is necessitated and the prognosis is poor. This may lead to an association of the hospital with poor outcomes and a belief that treatment will escalate the disease.^14^

## ADJUVANT THERAPY

As previously mentioned, with patients presenting at late stages, the role of surgery as a curative measure is significantly diminished. With locally advanced or metastatic disease, chemotherapy becomes ideal for treatment and palliation, yet the proper resources for its use are lacking, and its availability varies greatly across country and institution. Whereas patients in the West currently receive chemotherapy to downstage locally advanced tumors or treat node-positive and metastatic disease, adoption of this practice remains limited and varied on the African continent.

Hospitals in South Africa, Nigeria, and Cameroon report that more than 85% of their patients receive chemotherapy compared with 28% in Rwanda and 1.2% in Eritrea^6,10,12,16,17^ (Table 3). This disparity in chemotherapy rates reflects heavily on the available medical resources of the country. Rwanda has a shortage of health care providers who are equipped to treat patients with breast cancer, with only 15 general surgeons and no oncologists in the country.^6^ Women in Eritrea have few alternatives to surgical management, because the country has no permanent center for chemoradiotherapy treatment.^12^ Without the facilities or personnel trained to provide chemotherapy, these options are inaccessible for many patients. Treatment is skewed toward surgical options because of country-specific resource limitations, even when chemotherapy is recommended.

**Table 3:**
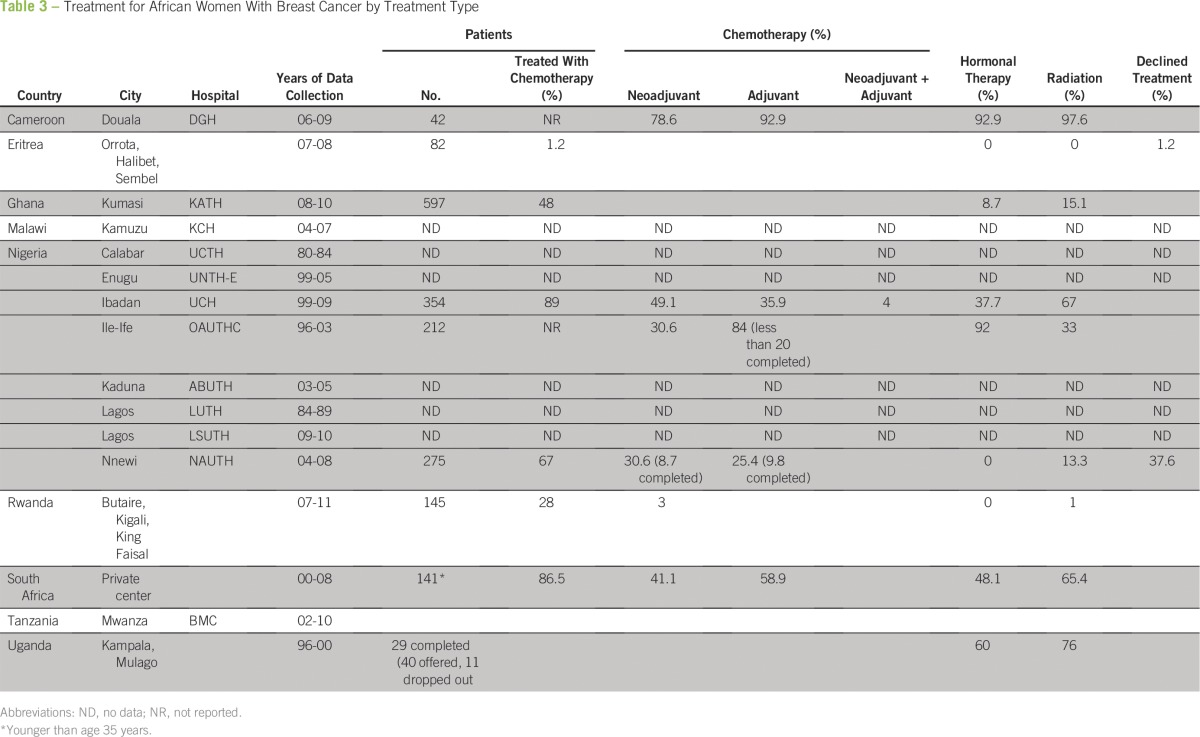
Treatment for African Women With Breast Cancer by Treatment Type

When chemotherapy is available, the majority of patients are not able to complete the recommended regimen, and treatment interruptions are common. A study from Uganda reports that only 29% of patients completed the six recommended cycles of chemotherapy, and fewer than 20% in Nigeria completed six to 12 cycles.^8,15^ The vast majority of patients (86.4%) report obstacles that interrupted or completely ended chemotherapy treatment.^7^ Financial cost of treatment represents the most prohibitive factor in continuing care.^14^ A course of chemotherapy costs approximately US$250, which most people are unable to pay.^15^ Furthermore, patients need to seek additional funds to pay for transportation to the hospital and for laboratory costs.^14^ Patients also report that they discontinue treatment because of a lack of space in the hospital and because they do not have a relative to care for them.

Radiation therapy is indicated to destroy localized breast cancer cells and reduce the rate of recurrence after surgical resection of the tumor.^19^ However, radiation therapy is not easily accessible, because only 23 of 52 African countries are equipped with radiation facilities.^3^ Rates for use of radiation therapy greatly differ among countries; 0% in Eritrea, 1% in Rwanda, 33.2% in Nigeria, 65.4% in South Africa, 76% in Uganda, and 97.62% in Cameroon.^6,8,10,12,15,17^ In countries in which radiation therapy exists, the facilities often attempt service beyond their capabilities, so the facilities become overcrowded with long wait times.^8^ Radiotherapy is often localized to private or tertiary hospitals, which limits its accessibility to many segments of the population.

In the absence of focused studies, tumor marker analysis is rarely possible in resource-poor countries.^12^ However, recent studies have suggested that the proportion of estrogen receptor–positive tumors in Africa is higher than previously reported (63% compared with 24%) and warrants further investigation.^20,21^ In the literature reviewed, only three studies reported the rate of hormone therapy use in their patients. Tamoxifen was used in 37.7% of patients in Nigeria, 48.1% in South Africa, 60% in Uganda, and 92.9% in Cameroon.^10,15-17^ Because hormone receptor status remains predominantly unknown, tamoxifen is currently prescribed blindly.^7^ Nevertheless, tamoxifen is cost prohibitive for most patients on the African continent, and newer classes of anti-estrogen aromatase inhibitors appropriate for postmenopausal women are not available or affordable in Africa.^8,15,17^

## FOLLOW-UP

Follow-up is poor among African patients with breast cancer, which hinders accurate assessment of the efficacy of treatment paths. Uganda and Cameroon report comparatively high follow-up rates: 28% of patients receive follow-up at the end of a 5-year period in Uganda, and 58.5% receive follow-up at 3 years in Cameroon.^10,15^ Studies from Nigeria show a more significant proportion of patients to be lost to follow-up, with only 37.9% of patients attending outpatient clinics 18 months after mastectomy; the percentage continued to decline until most patients were lost to follow-up by 30 months.^16^ The vast majority of patients are lost to follow-up within the first year of diagnosis, suggesting that interventions to improve follow-up should be implemented during the initial appointments.^8^

Survival rates are similarly poor, reflecting the advanced nature of the tumors at presentation and the numerous barriers to treatment. In South Africa and Nigeria, the 5-year survival rates are 20% and 14%, respectively.^4,17^ The prognosis predictably varies, depending on disease stage at presentation; 2-year survival in South Africa for those diagnosed as stage 0 to III is 56% compared with 16% for those in stage IV.^17^ Approximately 91.4% of deaths occur within the first year of diagnosis.^8^ Overall, studies generally assess the long-term outcomes of only those patients who completed treatment and, as a result, the survival rates are likely even worse than currently reported because they do not capture patients lost to follow-up or patients who did not complete treatment. The survival rate could be improved if interventions brought patients to clinical attention at earlier disease stages. Improvements in patient education and screening, including self-examination, that raise the awareness of breast cancer, the significance of breast lumps, and increased trust in the medical establishment could increase survival prognosis.

In conclusion, the goal of this review was to provide insight into the current management of breast cancer across the continent of Africa, focusing on surgical interventions. The literature shows that the rates of surgery, chemotherapy, and radiation therapy vary across different countries and institutions, emphasizing the need for a greater use of standardized cancer treatment guidelines. Surgery is the most commonly implemented therapy, but the intention of surgery remains consistently unclear, because treatment choice and disease stage do not correlate in regional studies. It seems that surgery is used as a treatment even in advanced disease because of the inaccessibility and expense of adjuvant chemotherapy and radiation therapy, and the high rates of patients lost to follow-up care.

To better understand the clinical reasoning for specific treatments and the efficacy of treatment options, it is necessary that medical records consistently include description of the disease and therapy. Improvements to follow-up monitoring would provide the much needed information on patient prognosis after treatment. Interventions to improve awareness of breast cancer and encourage self-examination, clinical breast examination, and prompt medical attention may improve survival. Adopting a minimum standard of care, including mastectomy, axillary clearance, and adjuvant therapy in resource-limited countries would be ideal.^22^ To this end, it would be most ideal if African women underwent resource-appropriate early detection examinations so they could present for surgical evaluation with resectable disease or have access to neoadjuvant chemotherapy to downstage locally advanced tumors and facilitate complete resection and closure.
